# Simple tone curves: theory and applications

**DOI:** 10.1007/s00371-026-04505-y

**Published:** 2026-05-18

**Authors:** James Bennett, Graham Finlayson

**Affiliations:** School of Computing Sciences, University of East Anglia, Norwich, NR4 7TJ UK

**Keywords:** Tone mapping, Tone curves, Image enhancement, Quadratic programming, Psychophysical experiment

## Abstract

A single tone curve that remaps the brightnesses of each image pixel is a simple and widely deployed way to enhance an image. Tone curves might be crafted by individual users or determined algorithmically in camera processing pipelines. The precise shape of the tone curve is not a priori strongly constrained, other than it is usually limited to increasing functions of brightness. In this paper, we constrain the shape further and define a tone curve to be simple if it has no or one inflexion point. With respect to our representation, wiggly tone curves have several inflexion points and are deemed to be complex. A key contribution of our work is to show how we can best approximate a complex curve with a simple counterpart. For the MIT-Adobe FiveK dataset, comprising thousands of images that are tone-adjusted by photographic experts, we calculate corresponding simple tone curve adjusted images. Using objective similarity metrics, we find that simple curves deliver equally good image enhancement. In terms of preference experiments, simple curves deliver slightly preferred images compared to complex counterparts. Similar results are reported for a second smaller underwater image dataset.

## Introduction

Tone curves map input tone values to corresponding output tone values by a continuous function, which is almost always increasing. These tone curves are used for many purposes. A tone curve that describes the mapping of the real-world scene radiance to measured pixel values is sometimes called a camera response function [1] and is often a near linear map. The inverses of these functions are also used to recover the scene radiance from an image. Tone curves are applied to map a large dynamic range of brightnesses that are measured at image capture to the lower dynamic range of a display [2]. Also, images are generally encoded after the application of a gamma function (a compressive tone curve function) because the target display inverts the gamma when an image is displayed. However, the predominant use of tone curves—and the one of interest in this paper—is image enhancement. To enhance an image, a tone curve is applied to the brightnesses of an input image which maps the brightness to output counterparts.

As discussed in [3], an enhancement is only suitable according to its purpose and audience; thus, there is no single optimal enhancement. However, regarding post-processing for photographic purposes, the hope and expectation are that the enhanced image is preferred compared to the original image. Many photographers routinely edit the photographs they capture to reproduce the tones of the image in a more pleasing manner. Significantly, whilst a tone curve can be described explicitly (by drawing a tone curve), tone adjustments are often implicit: users enhance images through manual slider-based adjustment, pre-made ‘filters’, or automatic enhancement tools. Often the effect of these adjustments can be modelled as the application of a tone curve. Perhaps unsurprisingly, [4] has shown that the ‘best’ tone-mapped image for one person may not lead to the most preferred image for another.

Tone maps can be applied to all colour channels in an image [5], to the individual colour channels [6, 7], or to a brightness channel only [8, 9]. In this last approach, whilst the adjustment takes place on a brightness channel, the effect is transferred to the colour image. For instance, we might map the brightness channel of an input image to an enhanced output brightness channel and then make sure the output image has a similar colour to the input. An example is an input image transformed to the CIELAB colour space [10] where the lightness channel, $L^*$, is tone mapped and then transformed back to RGB.

There are many techniques employed for enhancing images [11, 12]. The focus of this work is a global brightness remapping. An input brightness value $b_\textrm{in}$ is mapped to an output brightness value $b_\textrm{out}$ by the mapping function *T* as in

1 $$\begin{aligned} b_\textrm{out} = T(b_\textrm{in}). \end{aligned}$$

An enhanced image can be obtained by applying the mapping function (or tone curve) *T* to every pixel in an image. Take the scalar brightness input image $\mathcal {I}$ whose pixels are $\mathcal {I}(x,y)$ indexed by their spatial location *x* and *y*. Its enhanced counterpart is

2 $$\begin{aligned} \mathcal {O}(x,y) = T(\mathcal {I}(x,y)), \end{aligned}$$

when all *x* and *y* in the image are used. Note also that the inverse function $T^{-1}$ will recover the original brightness from the enhanced counterpart (ignoring quantisation and the possibility that two input brightnesses might be mapped to the same output). When the same function is applied to all pixels in the image, the enhancement is said to be *global*. When different functions are applied to different pixels, then the mapping would be *local*.

**Fig. 1:**
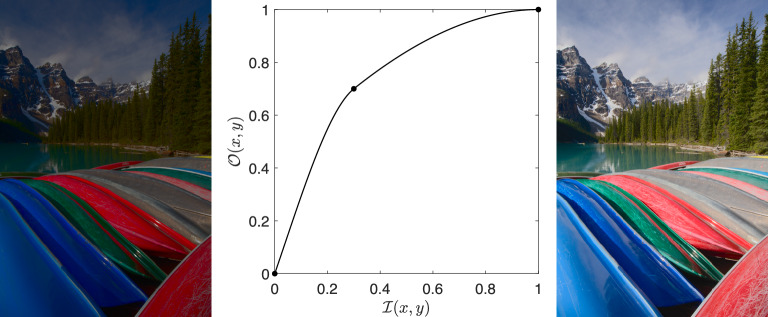
Left and right show, respectively, an input image and the result of applying the tone curve in the middle plot that maps the input brightness to output counterparts. The image is number 635 from the FiveK dataset with Expert E’s a* and b* channels

An example of tone mapping is shown in Fig. 1. An input image is shown on the left that produces the image on the right when enhanced with the tone curve shown in the middle. The brightness image, $\mathcal {I}$, is the $L^*$ channel in the CIELAB colour space normalised to [0, 1]. The curve depicts the enhancement *T* and is read that a pixel with a value of 0.3 in the input brightness image (marked as a ‘dot’ on the graph in the figure) would be mapped to a value of roughly 0.7 in the output image. The three points (0,0), (0.3,0.7), and (1,1) are interpolated with a cubic spline such that the gradient at the control point is continuous to form the smooth curve, passing through the control point as seen in Fig. 1. As discussed previously, to apply the tone mapping in this example we map the RGB image to CIELAB space, tone map $L^*$, and map back to RGB for display.

**Fig. 2:**
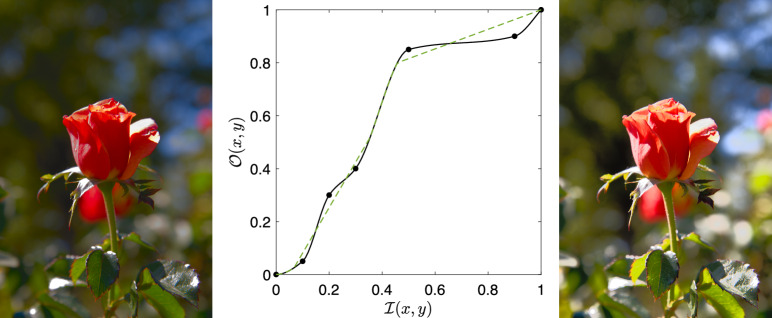
Left and right show, respectively, an input image and the result of applying the tone curve in the middle plot that maps the input brightness to output counterparts. The image is number 216 from the FiveK dataset with Expert A’s a* and b* channels. The green dashed line shows the simple curve approximation and is discussed later

A second example is shown in Fig. 2. Here, a user has chosen 5 control points and a tone curve is again created by spline interpolation. Clearly, the tone curve is ‘wiggly’ and in that sense complex. Informally, one of the questions we consider in this paper is whether complex (wiggly) tone curves are generally chosen by users to enhance their images. And, if they are chosen, whether they are advantageous from an image enhancement point of view compared to simpler counterparts.

In this paper, we propose that simple tone curves have the property that they have 0 or 1 inflexion points. For monotonically increasing tone curves (which account for almost all tone curves applied to images), this means the gradient of a tone curve might be monotonically increasing (e.g. $b_{out}=b_{in}^{2}$ has an increasing derivative) or it might increase to and inflexion point and then decrease as in an S-shaped curve. Or, the gradient might decrease monotonically (e.g. $b_{out}=b_{in}^{0.5}$) or decrease and then increase at the inflexion point (an inverse S-shaped curve).

The key concern of this paper is to consider the extent to which tone curves *T*, applied to images, are (or are not) simple. Accordingly, we seek an approximate tone curve $\hat{T}$, defined as

3 $$\begin{aligned} \hat{T} \approx T,\;\textit{where the shape of } \hat{T}  \textit{ is constrained to be simple}. \end{aligned}$$

Crucially, the algorithm we develop is optimal in the sense that it will find the closest counterpart to a complex curve over the set of all possible simple curves. The dotted line in Fig.  2 shows the closest simple tone curve approximation to the wiggly non-simple curve. We will consider the optimisation needed to solve this in detail.

Throughout this paper, we will generate tone curves according to two methodologies. The example in Fig. 1 shows tone mapping in $L^*$ (the brightness channel of the CIELAB colour space). Input RGB images are converted to the CIELAB space where the $L^*$ channel is tone mapped (either with a photographic expert’s tone curve or our simple tone curve approximation) and then converted back to RGB for image display. The second variant is the same except that the tone mapping relates the logarithm of the input brightnesses to the logarithm of the outputs. The output log $L^*$ values are exponentiated before we map CIELAB to RGB for display. We call our two brightness tone mapping strategies ‘$L^*$ tone mapping’ and ‘$\log (L^*)$ tone mapping’. Log-brightness encodings are widely adopted in models of human vision and in tone mapping, e.g. see the foundational work [13].

The tone-mapped image pairs of the MIT-Adobe FiveK dataset [8] (hereafter referred to as FiveK) are used to examine the extent to which complex curves can be approximated by simple counterparts. The dataset is comprised of 5000 images, each enhanced by 5 experts, meaning there are 25,000 pairs of input and corresponding tone-mapped output images. In the literature the input–output image pair is considered to be a single global tone curve apart. We found this to be approximately but not exactly true. Thus, many of the images used in our experiments are actually a small (visually imperceptible) delta from the actual images where a single tone curve does map inputs to outputs. In our experiments, we use the global expert tone curve and our simple $L^*$ tone curve approximation to render two output images, according to Eq. (2). We wished to compare these two images using a *metric* to determine the similarity of the photographic expert’s tone-adjusted image and the one rendered according to our simple approximation. According to [14, 15], if the mean CIELAB $\Delta E$ colour difference for an image pair is less than 3, then the image pair (for complex scenes of the type in the FiveK dataset) is almost visually indistinguishable. For the FiveK dataset, we find that a simple tone curve can, almost always, be used as a substitute for a complex curve. The same result is found in $\log (L^*)$ when we find the best tone curve in the log domain.

To further test the similarity or otherwise of simple variants to complex curves we ran a pairwise preference experiment. Observers were shown pairs of images and were simply asked which they prefer. In this experiment we chose 8 pairs with the highest colour difference ($\Delta E > 2.68$), 8 randomly chosen from above the 99th percentile ($0.78<\Delta E < 2.68$), and 8 randomly chosen from below it ($\Delta E < 0.78$). For each input image in our experiment, there are 3 output images: the expert-adjusted output and two simple tone curve variants for the $L^*$ and $\log (L^*)$ methodologies. To determine preference scores, Thurstone Case V analysis [16] was used to aggregate and scale the pairwise votes. Broadly, our experiments conclude that simple curves deliver almost the same enhancement as using the expert’s tone adjustment. If we solve for simple tone curves where brightnesses are encoded logarithmically, we actually find a slight preference for our simple curves.

For validation, we calculate the average CIELAB $\Delta E$ for the difference between actual and simple tone-adjusted images for the Gardline Underwater Tone Mapping dataset [17]. This image set comprises 60 underwater RGB images that have been tone mapped by three different underwater image analysts, where the global tone curves are supplied with the dataset. Again, we find images tone mapped with simple curves to match expert-adjusted images well. Significantly, for this dataset, a previous experiment [18] has *effectively* shown that simple curves are preferred over expert adjustments (though that work constrained curves to a particular parametric form that, serendipitously, can produce only simple curves).

Finally, we will consider the question whether a simple $L^*$ tone mapping recast in the log domain remains simple (and vice versa). We show, by example, that this is not the case. That is, it is possible that a simple curve in $L^*$ might be complex in $\log (L^*)$ and vice versa. Empirically, however, we find that it is rare for simple curves to become complex given a change in domain.

In the *Background* section, prior work related to the FiveK and Gardline datasets is discussed. The *Method* section contains how we obtain global tone-mapped images from the FiveK dataset, presents our algorithm for approximating any tone curve by a simpler proxy, and details the procedure of our psychophysical preference experiment. The *Results* section provides the outcome of the experiments, and the paper finishes with a *Discussion* followed by the *Conclusion*.

## Background

### MIT-Adobe FiveK dataset

The main dataset we will use is the FiveK dataset [8] which comprises 5000 photographs from a variety of scenes, including the natural world, people, the built environment, and man-made objects. Each photograph has been tonally retouched by five experts, resulting in 25,000 image pairs. Because each expert has edited according to their preference, the different renditions range from being similar to markedly different from each other.

The FiveK dataset is well established for tone mapping and image enhancement. It has been used in designing automatic image enhancement algorithms [19, 20], which are constrained to be global enhancements in [21] and further to be human-interpretable curves in [22]. When a tone mapping (or its parameters) is the algorithmic output [5–7, 23] (as opposed to the image itself), a tone curve can be determined from a thumbnail of an image and then applied to the full-resolution image to produce an enhanced output, reducing computational demand [24].

**Fig. 3:**
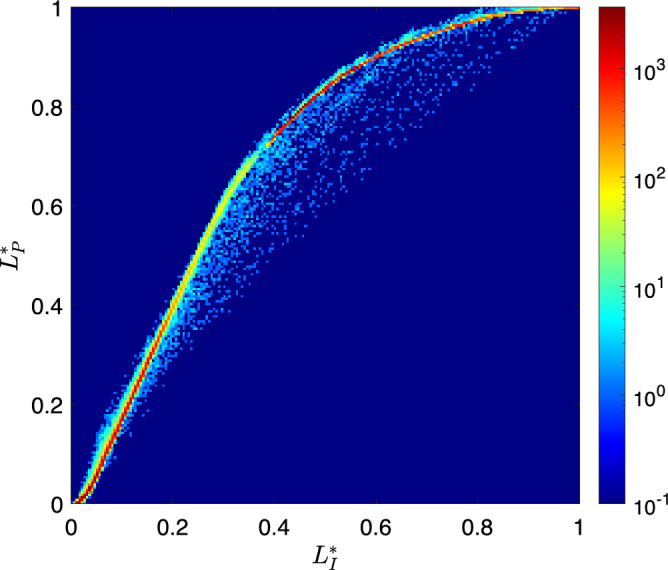
Scatter plot of $L*$ values of the input image against the expert’s rendition for image B3886. The colour of a point corresponds to the number of $L^*$ pairs at that point. The plot shows the image is not a perfect global transformation, but most points do lie on a global tone curve

Curiously, the premise behind the FiveK dataset—that there is a global tone curve to be learnt—turns out only to apply somewhat approximately in practice (at least for some images). In Fig.  3 we plot input (unedited) versus output (expert-adjusted) normalised CIELAB $L^*$ values [10] for an image. If the transformation was global, we would expect to see all points lie on a single curve. That is, every pixel of a given input brightness should map to the same output brightness.

In Fig. 3 most points lie on a curve, made apparent by the points coloured red. Of the remaining points, many lie close to this curve but do not map exactly, and several lie a long way from the curve. In this image (B3886), the tone mapping is not exactly global, but an underlying global tone curve can be seen for the majority of the pixels in the image. The goal of this work is to study global tone adjustments; therefore, we will outline how we accommodate the approximately global adjustments in the next section.

Of course, any edits made by a photographer will be made with respect to a particular colour space representation. Should we expect the input-lightness to adjusted-lightness plot to result in a one-to-one global curve? After all, if an image is tone mapped, say where the brightnesses were equal to (R+G+B)/3 (as opposed to Luminance) then the resulting lightness to lightness plot would not be global. From informally looking at this issue we found the $L^*$ channel often led to good global tone curves for the FiveK dataset and other colour encodings led to curves that were less global. This said, the exact colour space transforms used in mapping input to output images are not explicitly disclosed in the FiveK dataset.

Let us denote $\mathcal {I}$, $\mathcal {P}$ and $\mathcal {P}^{G}$, respectively, as an input image, an expert-adjusted output, and a global approximation thereof. Considering an image as a function whose arguments, *x* and *y*, are the location of the pixel, $\mathcal {I}(x,y)$, $\mathcal {P}(x,y)$, and $\mathcal {P}^{G}(x,y)$ yield 3-vectors for a single given pixel. For the purposes of this paper, the relationship between these three images is,

4 $$\begin{aligned} \begin{array}{l} \mathcal {I}(x,y)=[L^*_I\;a^*_I\;b^*_I]^\top \\ \mathcal {P}(x,y)=[L^*_P\;a^*_P\;b^*_P]^\top \\ \mathcal {P}^{G}(x,y)=[T(L^*_I)\;a^*_P\;b^*_P]^\top \end{array} \end{aligned}$$

where here and throughout the superscript $^\top$ denotes the transpose operator and *T* is a global tone mapping function such that $T(L^*_I) \approx L^*_P$. For our purposes, we find the best *T* using matching [3], pairing quantiles of $L^*_I$ to the same quantiles of $L^*_P$.

**Fig. 4:**
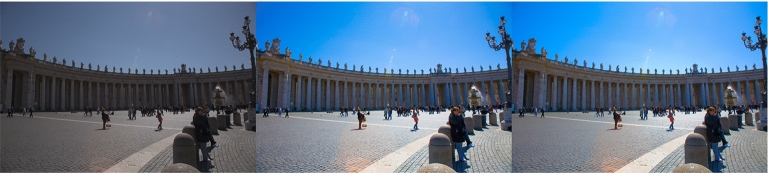
Left, middle, and right show, respectively, an input image, $\mathcal {I}$; the expert’s rendition, $\mathcal {P}$; and the global approximation, $\mathcal {P}^{G}$. In this example, image 1925 and Expert C are shown

Of course, we should only use this approach if, visually, $\mathcal {P}^{G}$ looks like $\mathcal {P}$. To test this, we calculate the mean CIELAB $\Delta E$ colour difference for the 25,000 images (5000 input images adjusted for preference by 5 experts) in the FiveK dataset. 50% have a mean $\Delta E$ of less than 0.36 and even the 0.99 quantile is only 1.88. It has been proposed that if a pair of images have a mean $\Delta E$ of less than 3, then the image pair is visually indistinguishable [14, 15]. We found this to be the case for 99.9% of our data. The maximum mean $\Delta E$ is 10.1, but this is an outlier; all of the remaining 24,999 images in the FiveK dataset have a $\Delta E$ less than 4. Indeed, Fig.  4 shows the input image $\mathcal {I}$, the expert adjusted output $\mathcal {P}$, and our global approximation $\mathcal {P}^{G}$ for the second maximal case where $\Delta E$ of 3.94. Even here, the images $\mathcal {P}$ and $\mathcal {P}^{G}$ look very similar. Henceforth, we use $\mathcal {P}^{G}$ as our ground truth.

### Applying enhancements to images

The tone curve function in Eq. (4) may well be wiggly. In the next section we show how to determine a simple (zero or one inflexion point) variant which we denote $\hat{T}$. When applied to an input image, $\mathcal {I}$, an output $\hat{\mathcal {P}}$ is generated. Adopting the notation of Eq. (4), the corresponding simple tone-mapped image, $\hat{\mathcal {P}}(x,y)$ is defined as

5 $$\begin{aligned} \hat{\mathcal {P}}(x,y)=[\hat{T}(L_I^*)\;a^*_P\;b^*_P]^\top . \end{aligned}$$

### Gardline underwater tone mapping dataset

For validation, we use a second dataset that contains 60 underwater RGB images have been tone mapped by three different underwater image analysts resulting in 180 image pairs and corresponding tone curves. The analysts manipulated these images to make details as conspicuous as possible rather than to make the images preferred [17]. Advantageously, these image are exactly global tone adjustments.

## Method

### Obtaining brightness images

The tone adjustments investigated in this work are global brightness remappings; thus, we require brightness images from the dataset. The FiveK dataset images can be downloaded individually or as an Adobe Lightroom catalogue. There are 5000 input images and 5 output counterparts for each input (the same image manipulated by 5 experts). Adopting the procedure of [23] (also used by [6, 21]), the collection *Input/Input with Daylight WhiteBalance minus 1.5* is used as the input images and image pairs are exported from Adobe Lightroom in ProPhotoRGB as 16-bit TIFF images with the longest edge sized to 640 pixels.

In our experiments, conducted in MATLAB, each TIFF image is read as a 16-bit unsigned integer and normalised so the brightest pixel on any RGB channel is equal to 65535. Then, each image is converted from ProPhotoRGB into the CIELAB colourspace and $L^*$ values are divided by 100, so the brightness image is in the range [0, 1].

Some images were found to have spurious $L^*$ brightnesses causing undue influence on the tone curve. Excluding saturated pixels with a value of 0 or 1, the 0.001 and 0.999 quantiles of the brightness image are found. All pixels with a value above the 0.999 quantile value are set to that value and equivalently for all pixels below the 0.001 quantile value. The brightness image has now been limited to the range between the 0.001 and 0.999 quantile values. Using the thousandth quantiles has an inconsequential effect on the image perception but mitigates the spurious brightnesses affecting the tone curve.

In the introduction, we explained that one may wish to encode the $L^*$ brightness image and stated we will consider two methodologies: ‘$L^*$ tone mapping’ and ‘$\log (L^*)$ tone mapping’. Here we detail how we transform to the $\log (L^*)$ encoded brightness space.

From our tone curve definition in Eq. (1), where the dash denotes transformed quantities, we transform to our encoded space using the encoding function *g*,

6 $$\begin{aligned} b_\textrm{in}'&= g(b_\textrm{in}), \end{aligned}$$

7 $$\begin{aligned} b_\textrm{out}'&= g(b_\textrm{out}). \end{aligned}$$

In transformed space, the tone curve, $T'$, is the mapping from input encoded brightnesses to output encoded brightnesses and is described as,

8 $$\begin{aligned} b_\textrm{out}' = T'(b_\textrm{in}'). \end{aligned}$$

We choose the natural logarithm as our transformation, though others can be used. Since the log of 0 cannot be taken, any zero values are set to the next smallest value of $\frac{1}{2^{16}-1}$. In essence, the transformation function is simply, $g: b \mapsto \ln (b)$, for input or output brightnesses *b*. However, we want to linearly rescale to the range [0, 1]; therefore, our *g* becomes

9 $$\begin{aligned} g: b \mapsto 1 - \frac{\ln (b)}{\ln (\min (b))}. \end{aligned}$$

In the next steps we will model the tone mapping from input to output brightnesses. By design, the following steps to extract and simplify the tone curve are indifferent to the encoding of brightnesses. Hence, the notation is common and we now have the images $\mathcal {I}$ and $\mathcal {P}$ according to the notation of Eq. (4).

The global tone curves that produce the images $\mathcal {P}^{G}$ are extracted by matching the quantiles of the $L^*_I$ image to the $L^*_P$ image. These quantiles define control points which are interpolated to yield the global tone curve *T*.

### Discrete tone curve representation

Up to this point, tone curves had been described as per Eq. (1) where a function *T* maps an input brightness value $b_\textrm{in}$ to an output brightness value $b_\textrm{out}$. Let us now represent a tone curve function using a vector representation. Suppose we have *n* uniformly spaced input brightness values in the domain [0,1], written as the *n*-component vector $\boldsymbol{b}=[0,1/(n-1),2/(n-1), \ldots ,1]^\top$. Evaluating the tone curve *T* from Equation (1) at these input brightness values, results in the *n*-vector $\boldsymbol{t}=[T(0),T(1/(n-1)),T(2/(n-1)), \ldots ,T(1)]^\top$. We use $t_i$ to denote the *i*-th component of $\boldsymbol{t}$. Pairing the components of $\boldsymbol{b}$ and $\boldsymbol{t}$ yields a sequence of control points

10 $$\begin{aligned} (b_1, t_1), (b_2, t_2), \ldots , (b_n, t_n) \end{aligned}$$

which, with an appropriate interpolation scheme, define the function *T* such that $t_i = T(b_i)$. Accordingly, we assume the equivalence,

11 $$\begin{aligned} \boldsymbol{t}\equiv T. \end{aligned}$$

In our experiments, we use $n=100$ and interpolate with shape-preserving monotone Piecewise Cubic Hermite Interpolating Polynomials (PCHIP) [25]. Thus, for the 100 uniformly spaced input $L^*$ values between the minimum and maximum brightness image value, we calculate the outputs of the tone curve as the 100-vector $\boldsymbol{t}$.

### Simplifying tone curves

Tone curves *T* are increasing functions—that is, if $b_j\ge b_i$ then $T(b_j)\ge T(b_i)$. As we discussed in the introduction, we have an expectation that tone curves should additionally be *simple* in some sense. We do not expect the tone curve to be wiggly, as in the example of Fig. 2 in the introduction. Wiggly here, intuitively, means that there are only so many ‘turns’ in the tone curve; the more it looks like a series of steps the more wiggly it is. In part, we feel that wiggly tone curves should not be used because, from the user’s viewpoint, they seem to be difficult to define (and if they can be defined they may be difficult to replicate).

**Fig. 5:**
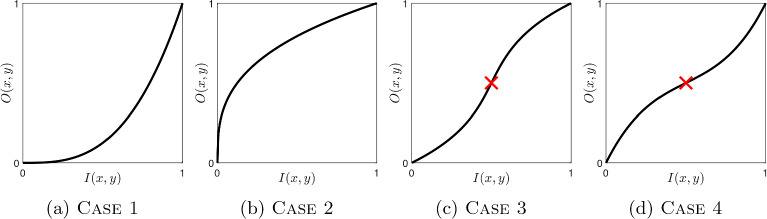
Depiction of the four simple cases

We define simple tone curves as increasing functions that have no or only a single inflexion point. An inflexion point occurs where the curvature changes sign or equivalently a zero crossing of the second derivative. For a given tone curve represented by a vector $\boldsymbol{t}$—which may have more than one inflexion point—we would like to find an approximate tone curve $\boldsymbol{\hat{t}}$ that is simple and as close as possible to $\boldsymbol{t}$. Figure 5 depicts exemplars for the four cases allowed by this definition.

In Sections 3.3.1 through 3.3.4 we write down the constraints we will need to solve for a simple curve that is closest to a complex counterpart and we also present the optimisation that needs to be solved.

#### Monotonic increasing function constraints

An increasing function means each point on the curve is greater than or equal to the last $\hat{t}_i \ge \hat{t}_{i-1}$, or in this context, each output brightness must be brighter than the previous. Thus we require,

12 $$\begin{aligned} \hat{t}_i - \hat{t}_{i-1} \ge 0 \quad \textrm{for} \; i = 2,3, \ldots ,n. \end{aligned}$$

For the purposes of our optimisation, it is useful to write our constraints in vector–matrix form. Let us define an $(n-1)\times n$-sized matrix $\boldsymbol{D}$,

13 $$\begin{aligned} \boldsymbol{D} \triangleq \begin{bmatrix} -1 & \quad 1 & \quad 0 & \quad \cdots & \quad 0 & \quad 0 & \quad 0 \\ 0 & \quad -1 & \quad 1 & \quad \cdots & \quad 0 & \quad 0 & \quad 0 \\ \vdots & \quad \vdots & \quad \vdots & \quad \ddots & \quad \vdots &  \quad \vdots & \quad \vdots \\ 0 & \quad 0 & \quad 0 & \quad \cdots & \quad -1 & \quad 1 & \quad 0 \\ 0 & \quad 0 & \quad 0 & \quad \cdots & \quad 0 & \quad -1 & \quad 1 \end{bmatrix}, \end{aligned}$$

where $\boldsymbol{D}$ acts as an operator on $\boldsymbol{\hat{t}}$ to calculate the discrete derivative such that,

14 $$\begin{aligned} {[}\boldsymbol{D}\boldsymbol{\hat{t}}]_i = \hat{t}_i - \hat{t}_{i-1}, \quad \textrm{for} \;i=2,3, \ldots ,n . \end{aligned}$$

Equation (14) calculates a first-order backward finite difference approximation, which is a discrete calculation for the gradient of the tone curve. Due to the backward difference, we note that the result is undefined for $i=1$. Any tone curve should have the property $[\boldsymbol{D}\boldsymbol{\hat{t}}]_i \ge 0$.

#### Inflexion point constraints

Let us define the operator $\boldsymbol{D}^2$ that acts on a tone curve vector to calculate the discrete second derivative,

15 $$\begin{aligned} \boldsymbol{D}^2 \triangleq \begin{bmatrix} 1 &  -2 &  1 &  0 &  \cdots &  0 &  0 &  0 &  0 \\ 0 &  1 &  -2 &  1 &  \cdots &  0 &  0 &  0 &  0\\ \vdots &  \vdots &  \vdots & \vdots &  \ddots &  \vdots &  \vdots &  \vdots &  \vdots \\ 0 &  0 &  0 &  0 &  \cdots &  1 &  -2 &  1 &  0 \\ 0 &  0 &  0 &  0 &  \cdots &  0 &  1 &  -2 &  1 \end{bmatrix}, \end{aligned}$$

Here, $\boldsymbol{D}^2$ is not defined for either boundary element; thus, the size of $\boldsymbol{D}^2$ is $(n-2)\times n$. When $\boldsymbol{D}^2$ operates on a vector $\boldsymbol{\hat{t}}$, it calculates the finite second-order central finite difference approximation such that,

16 $$\begin{aligned} {[}\boldsymbol{D}^2\boldsymbol{\hat{t}}]_i = \hat{t}_{i-1} -2\hat{t}_{i} + \hat{t}_{i+1}, \quad \textrm{for} \;i=2,3, \ldots ,n-1. \end{aligned}$$

Now, corresponding to Fig. 5, we can write down the required second derivative constraints.

In Case 1 (Fig. 5a ) the second derivative is entirely positive, therefore,

17 $$\begin{aligned} {[}\boldsymbol{D}^2\boldsymbol{t}]_i \ge 0 \quad \textrm{for} \;i=2,3, \ldots ,n-1, \end{aligned}$$

In Case 2 (Fig. 5b ) the second derivative is entirely negative, therefore,

18 $$\begin{aligned} {[}\boldsymbol{D}^2\boldsymbol{t}]_i \le 0 \quad \textrm{for} \;i=2,3, \ldots ,n-1, \end{aligned}$$

In Case 3 (Fig. 5c ) the second derivative is positive up to an inflexion point, *f*, and then negative. 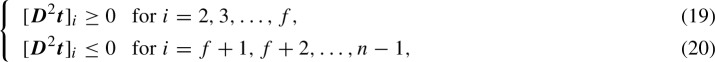


In Case 4 (Fig. 5d ) the second derivative is negative up to an inflexion point, *f*, and then positive. 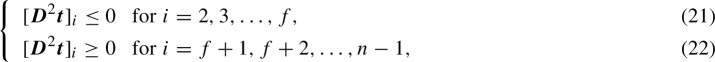


To ease notation, we define the **restriction** matrices $\boldsymbol{R}^{c,f}$ that are indexed by the case number $c \; \in \; \{1,2,3,4\}$ and the inflexion point $f \; \in \; [2..n-1]$, where there is no superscript $^f$ for Case 1 and Case 2 curves. For each case, of simple curves we place a different restriction on the shape of tone curve we seek.

The restriction matrix for Case 1 is

23 $$\begin{aligned} \boldsymbol{R}^1 = \boldsymbol{D}^2. \end{aligned}$$

The Case 3 curves depend also on the inflexion point, therefore

24 $$\begin{aligned} \boldsymbol{R}^{3,f} = \begin{bmatrix} \boldsymbol{D}^2 _{[2..f]} \\ -\boldsymbol{D}^2 _{[(f+1)..(n-1)]} \end{bmatrix} \quad \textrm{for} \;f=2,3, \ldots ,n-1. \end{aligned}$$

where block notation is used to represent the vertical concatenation of the matrices.

Since Case 2 is the reverse of Case 1 and Case 4 the reverse of Case 3, we write

25 $$\begin{aligned} \boldsymbol{R}^2&= - \boldsymbol{R}^1, \end{aligned}$$

26 $$\begin{aligned} \boldsymbol{R}^{4,f}&= - \boldsymbol{R}^{3,f}. \end{aligned}$$

It will be convenient to write the optimisation as a search over *c* (the case) and *f* (the inflexion point). Of course, Case 1 and Case 2 have no inflexion point. However, for convenience, we define$\boldsymbol{R}^{1,f}=\boldsymbol{R}^1$ and $\boldsymbol{R}^{2,f}=\boldsymbol{R}^2$.

#### Tone curve interval constraints

Finally, images have a dynamic range from the darkest pixel value to the brightest one. We want to map to the same dynamic range as the expert, so we constrain the first and last points of the curve to be equal. We require

27 $$\begin{aligned} \hat{t}_1&= t_1 , \end{aligned}$$

28 $$\begin{aligned} \hat{t}_n&= t_n , \end{aligned}$$

which is written in matrix vector form as

29 $$\begin{aligned} \boldsymbol{B}\boldsymbol{\hat{t}} = [t_1\;t_n]^\top , \end{aligned}$$

where $\boldsymbol{B}$ is an $2 \times n$ matrix with $\boldsymbol{B}_{1,1} = 1$, $\boldsymbol{B}_{2,n} = 1$, and 0 elsewhere.

#### Solving for the best simple tone curve approximation

We have a target tone curve $\boldsymbol{t}$ and the constraints that $\boldsymbol{\hat{t}}$ must satisfy in order to be simple. Now we can write down the optimisation to find the best simple curve. 
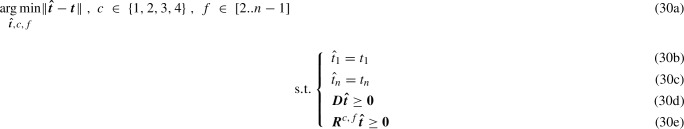


For each *c* and *f*, Eq. (30) is a quadratic programming problem: a quadratic objective is minimised subject to constraints [26, 27]. Quadratic programmes are readily solved and the solution is a global optimum. Here, we effectively search over all cases and inflexion points and the best overall curve is found. Because the restriction matrices for Case 1 and 2 have a *null* index $^f$ (these cases have no inflexion point, therefore $^f$ is meaningless), we do not have to search *f* for these two cases.

### Psychophysical experiment

We conduct a psychophysical experiment, evaluating human preference towards the ground truth image and the simplified one. In addition to evaluating subjective similarity, if the images are not deemed similar we can assess which one is preferred. Simple curves may produce images that are subjectively preferred that is not captured by the objective metrics. In doing so, we are asking whether observers prefer simply enhanced images to complexly enhanced ones.

For this experiment, a subset of 24 images from the FiveK dataset were used. These images were the worst 8 images as ranked by the mean $\Delta E$ scores from the $L^*$ tone mapping methodology, a random set of 8 above the 99th percentile, and a random set of 8 from below the 99th percentile. Four renditions of each image were tested: the input image $\mathcal {I}$, the ground truth expert image $\mathcal {P}^{G}$, and the simply enhanced images $\mathcal {\hat{P}}$ in both $L^*$ and $\log (L^*)$ methodologies.

The type of experiment conducted was a force-choice pairwise comparison experiment. The observer was shown pairs of images on a calibrated computer monitor where *image A* on the left will be one of the four renditions and *image B* on the right will be another. The observer is asked which image they prefer and they respond—they are forced to choose one image or the other—by clicking a button on the computer application. The observer is only shown one pair at a time, and for 4 renditions, there are 6 pair combinations. The user is shown each pair twice, reversing A and B so there are 12 comparisons per image, resulting in 288 comparisons per observer. The forced-choice pairwise comparison experiment design was chosen because it has been shown to result in the smallest measurement variance which provides the most accurate results [28]. The design is straightforward and does not require teaching naïve observers how to judge images which risks biasing the results.

Furthermore, this two-alternative forced-choice paradigm for judging preference is commonly used in the literature. In [29], a dataset was collected for subjective quality assessment of four tone mapping operators. Observers were asked to choose the image they prefer in a forced-choice pairwise comparison experiment. The experiment was large scale, with around seventy unique observations per pair. In [30], a forced-choice paradigm with a reference patch is used to validate deep features as a perceptual similarity metric. Perceptual quality of tone-mapped images has been compared using pairwise comparison in both early HDR work [31] and more recently with ten observers [32]. Notably, recent work [33] has used pairwise comparison on the FiveK dataset to learn about subjective image preferences. Previous work [34] comparing web-based preference experiments to those undertaken in laboratory conditions has shown the two-alternative forced-choice method to produce results with high intra-observer consistency and high agreement between observers. It was shown in their experiment that Thurstonian scaled values converged after approximately 500 total pairwise comparisons, supporting the viability of small-scale forced-choice experiments.

Our psychophysical experiment was conducted according to Recommendation ITU-R BT.500-15 [35]. There were 8 naïve observers who had colour normal vision, confirmed by testing with the Ishihara plates [36], and the experiments were conducted in a grey room with controlled lighting and a calibrated computer monitor. The images are shown in random order, restricted to not showing the same image simultaneously. The images were displayed in the colour profile of the monitor and the user voted via a MATLAB graphical user interface.

The results of the pairwise comparison experiment are a series of votes by each observer for each image. To compare these to each other, psychometric scaling is performed under Thurstone Case V assumptions [16] using the pairwise comparison toolbox of Pérez-Ortiz and Mantiuk [37], which provides maximum likelihood estimation with a regularisation prior that reduces estimation error when the number of observers is small. Psychometric scaling converts these votes into Just Objectionable Difference (JOD) units where higher is better and 1 JOD corresponds to 75% discrimination threshold. The units are arbitrary and cannot be compared to other experiments, but the relative differences are meaningful and allow all pairwise tests to be compared on the same scale.

## Results

### Objective evaluation of simple enhancements

The key concern of this paper is to consider to what extent tone curves applied to images are or are not simple (see Sect.  3.3 for definition). Using Eq. (30), we solve for the simple curve $\hat{T}$ which is optimally close to *T*, but simple. When $\hat{T}$ is applied to $\mathcal {I}$ it yields $\hat{\mathcal {P}}$ as per Eq. (5). For all 25,000 expert tone mappings in the FiveK dataset, we obtain the best simple tone curve for the brightness images using the $L^*$ and $\log (L^*)$ methodologies.

**Fig. 6:**
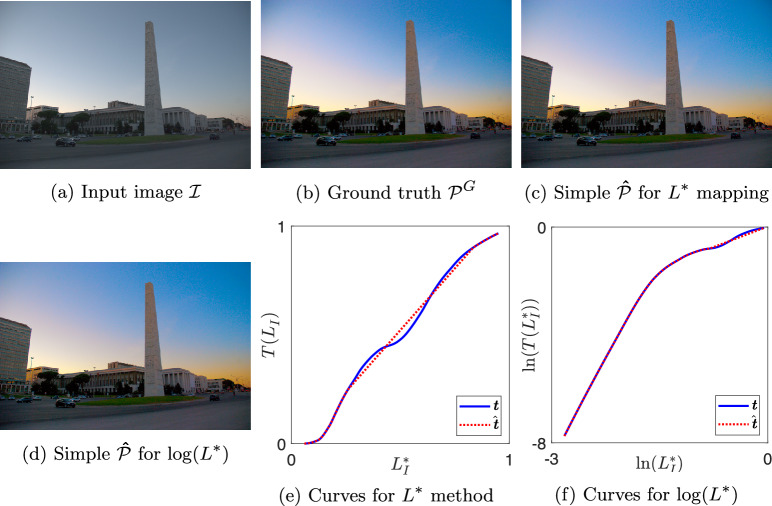
Result images and tone curves for image 4215 by expert C

**Fig. 7:**
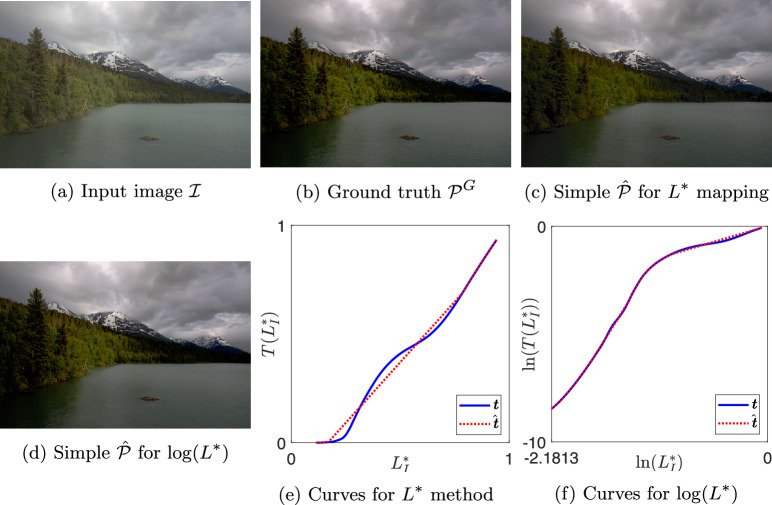
Result images and tone curves for image 2049 by expert A

**Fig. 8:**
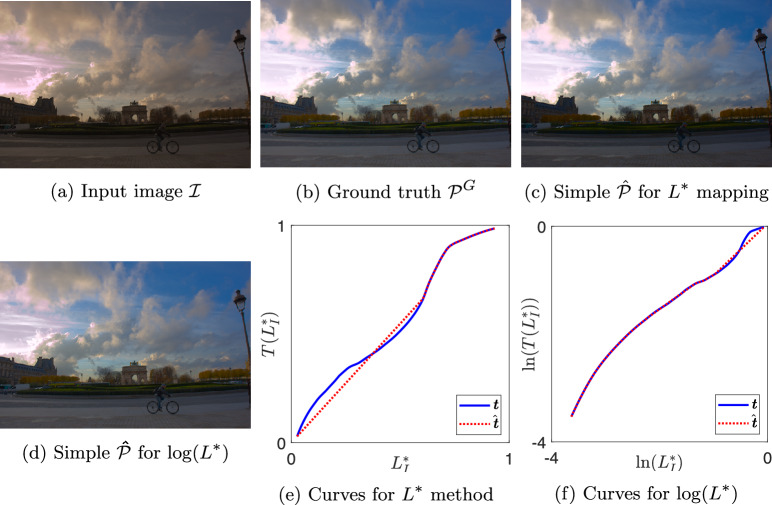
Result images and tone curves for image 2823 by expert A

**Fig. 9:**
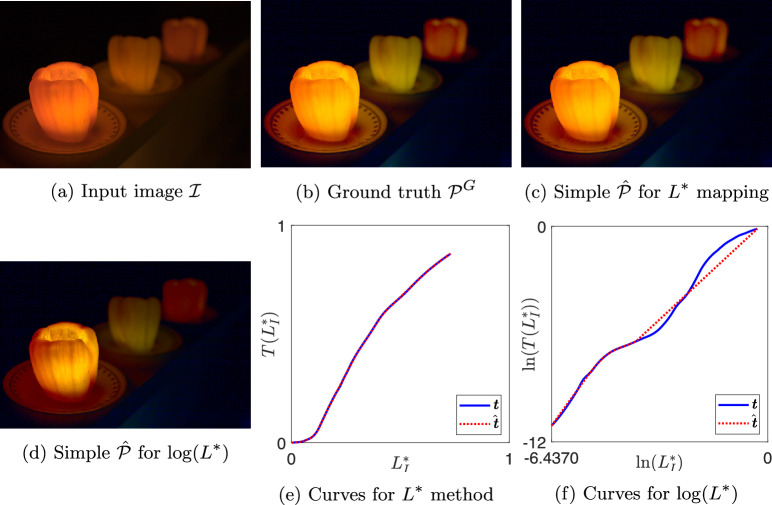
Result images and tone curves for image 3123 by expert C

In Figures 6, 7, 8, and 9 we show examples of our simple tone curve approximation approach. Our aggregate results will show that the majority of our solution curves are numerically close and appear visually similar to the expert curves, producing images that are imperceptibly different. This result tells us that experts mostly use simple (or close to simple) tone curves. We instead show examples where the colour difference to ground truth is in the worst one per cent and the solution curves differ significantly from the expert curves. However, it is evident from the figures that when a single curve in $L^*$ is not a good approximation, the simple curve in $\log (L^*)$ well approximates the expert adjustment or vice versa. The figures are shown here as a visual example of how our method operates. We will revisit these examples in the Discussion section for a targeted analysis of the successes and limitations.

Each figure shows one expert’s enhancement on one image and our simple approximations. Sub-figures (a) to (d) show the example images where (a) is the input image $\mathcal {I}$ and (b) is the ground truth image $\mathcal {P}^{G}$. Image (c) is the output generated using a simple curve mapping $L^*$. Finally, (d) is the output where the best simple curve approximation is found in $\log (L^*)$ space. In sub-figures (e) and (f), we plot the global tone curves for $L^*$ and $\log (L^*)$, respectively. In both cases the non-simple ground truth is shown in solid blue and the best simple approximation in dotted red. Table 1 shows the colour difference between images $\mathcal {\hat{P}}$ and $\mathcal {P}^{G}$ for both methodologies, where the value ranks out of the 25,000 tone curves.

**Table 1:** Mean $\Delta E$ and rank between simple $\mathcal {\hat{P}}$ and ground truth $\mathcal {P}^{G}$ images for examples in Figs. 6 to 9

	$L^*$		$\log (L^*)$	
Image	$\Delta E$	Rank	$\Delta E$	Rank
C4215	1.62	24,957	2.01	24,949
A2049	3.12	24,998	2.14	24,957
A2823	3.13	24,999	1.92	24,942
C3123	0.0406	18,176	5.60	24,997

Now, let us consider how well a simple tone curve can approximate the ground truth for the whole FiveK dataset. For each of the 5 experts, over their 5000 edits, we calculate the mean $\Delta E$ between the simple tone curve approximation and the ground truth. Thus, for each expert we have 5000 mean $\Delta E$ (image difference) scores. For these scores we can calculate the median $\Delta E$ (0.5 quantile) an other quantiles of interest. We calculate the errors for the quantiles 0.5, 0.9, 0.95, 0.99, and 1, and these are tabulated in Tables 2 and 3 for FiveK in $L^*$ and $\log (L^*)$ encodings.

For each FiveK expert in $L^*$ encoding, the 0.99 mean $\Delta E$ quantile is close to 1 or below where a value less than 1 is considered to be an imperceptible difference. Therefore we can say at least 99% of the dataset is very well approximated by a simple tone curve adjustment. The worst single adjustment for each expert ranges from 2.03 to 3.22 where a difference less than 3 is not immediately noticeable, so we approximate these reasonably well also. The FiveK dataset in $\log (L^*)$ encoding has more varied results. The worst $\Delta E$ for expert B is 2.6 which is less than the 2.88 in $L^*$ encoding, but the worst single tone mapping in log encoding has a $\Delta E$ of 8.02 which is greater than in $L^*$ encoding. The 0.99 quantile results for log space show that the majority of curves are also very well approximated by a simple curve in our $\log (L^*)$ encoding.

**Table 2:** Quantiles of the mean $\Delta E$ per FiveK expert

	Quantiles of mean $\Delta E$				
Expert	0.50	0.90	0.95	0.99	1.00
FiveK A	0.0159	0.189	0.341	1.09	3.22
FiveK B	0.0163	0.138	0.212	0.519	2.88
FiveK C	0.0171	0.226	0.411	0.966	2.54
FiveK D	0.0063	0.098	0.179	0.543	2.03
FiveK E	0.0083	0.0747	0.148	0.569	2.29

**Table 3:** Quantiles of the mean $\Delta E$ per FiveK expert in log space

	Quantiles of mean $\Delta E$				
Expert	0.50	0.90	0.95	0.99	1.00
FiveK A	0.017	0.34	0.657	1.62	6.90
FiveK B	0.0168	0.372	0.551	0.93	2.60
FiveK C	0.0222	0.247	0.427	1.11	5.60
FiveK D	0.0094	0.181	0.378	1.02	8.02
FiveK E	0.0135	0.14	0.261	0.722	3.19

The edits that experts made to images can vary significantly. Thus, for each input image, we calculate the *median* across the 5 mean $\Delta E$ values for the 5 expert-adjusted outputs. We report the quantile errors for these median adjustments in Table 4. Considering the median result is informative: if all experts needed to make complex enhancements for an image to look reasonable—and those enhancements could not be well approximated by a simple curve—then the maximum median $\Delta E$ would be high. Instead, we see that these are generally low. This suggests that any high value in the per-expert results is likely due to an individual expert using a wiggly curve on one occasion rather than that image inherently requiring a complex adjustment. More specifically, the FiveK maximum median error for the FiveK dataset is slightly more than one which is almost an imperceptible difference. Additionally, the maximal median error for the log-space FiveK curves is considerably lower than any per-expert maximum, mitigating some of the higher and varied results we saw from the per-expert $\Delta E$ values.

**Table 4:** Quantiles of the $\Delta E$ values for the median adjustment

	Quantiles of mean $\Delta E$				
Dataset	0.50	0.90	0.95	0.99	1.00
FiveK	0.0106	0.0695	0.112	0.279	1.09
FiveK log	0.0149	0.119	0.206	0.425	1.34

**Fig. 10:**
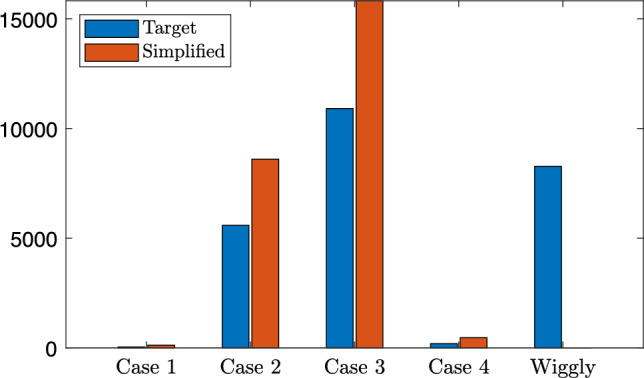
Classification of curve type from FiveK dataset in $L^*$ encoding before and after finding simple curve approximations with our method

Finally, it is interesting to objectively consider the proportion of curves that were simple or complex and the cases to which they have been simplified. The number of inflexion points is calculated by counting zero crossings of the second derivative. We use the finite second-order central finite difference approximation, here not omitting the step size. In practice we find that almost every expert curve is wiggly due to very small inflexions in the curve that arise from the discrete nature and interpolating polynomial but are not readily observable or have a meaningful impact on the curve. As such, we treat second derivatives less than $2 \times 10^{-3}$ as noise and round to 0. We then classify the curves into wiggly or the four simple cases by counting the number and direction of zero crossings. The results are presented in Fig.  10.

In blue we show the number of Case 1 through 4 tone curves found for the 25,000 adjustments made by our experts. Perhaps unsurprisingly, Case 1 is hardly chosen. Referring back to Fig. 5, in Case 1 images are made darker, whereas in general images get brighter in tone adjustment. In Case 2 we have something that looks either like a fractional gamma (used for encoding images) or a typical *camera curve*. These are about 20% of the adjustments. The most common adjustment is Case 3 which effectively stretch contrast in a part of the brightness range and, respectively, compress brightnesses near black and white, i.e. they are, at least abstractly, S-shaped curves. The Case 4 curves are rarely used as these will reduce contrast for mid tones (and enhancements generally increase contrast). About 30% of the adjustments are wiggly.

In red we show the distribution of the best simple approximations. Of course there are now no wiggly curves and we see that these have mostly been mapped to the Case 2 and 3. So, one conclusion to draw from Fig. 10 is that almost all of the simple curve approximations for the 25,000 images are either Case 2 (no inflexion point, decreasing derivative) or Case 3 (one inflexion point, increasing then decreasing derivative).

### Subjective evaluation of simple enhancements

**Fig. 11:**
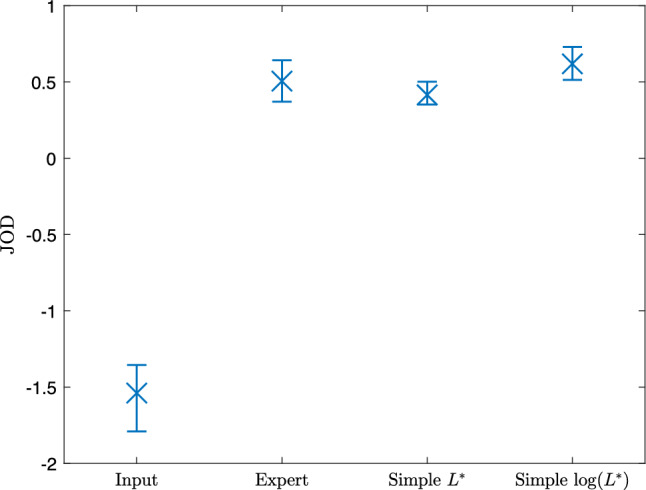
Psychometric scaling of pairwise preference experiment

In the previous experiment, the simplifying method was applied to each image and we found, objectively, the images to be similar. We wish to evaluate this subjectively also. Figure 11 shows the scaled results where the error bars denote 95% confidence intervals computed by bootstrapping with 1000 samples.

As expected, users prefer the expert and simple renditions to the input, but it serves a useful baseline. The expert, simple, and log-space simple renditions all have similar JOD values and their confidence intervals are overlapping. This is an important result and tells us that the observers do not exhibit a preference for any of these renditions; therefore, we conclude that the solved-for simple tone curve well approximates the expert tone adjustments. A point of interest is the greater preference of the simple curves solved for in log space. We must be careful about drawing conclusions with the overlapping confidence intervals, but despite the slightly higher $\Delta E$ values, for the images in this experiment the results suggest users prefer the simple curves solved in log space. Another point of interest is that whilst the simple rendition is slightly below the expert rendition, indicating a slight preference to the expert images, the log-space simple rendition is greater than the expert rendition suggesting in fact that these observers prefer a simple tone enhancement to the expert tone enhancement.

### Gardline dataset

**Fig. 12:**
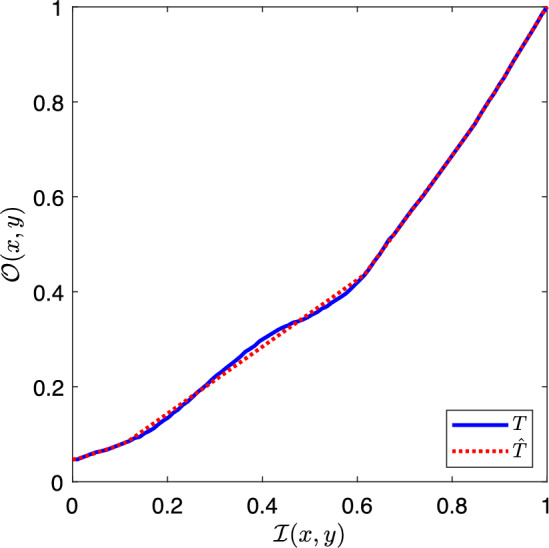
Expert and simple tone curves for images in Fig.  13

**Fig. 13:**
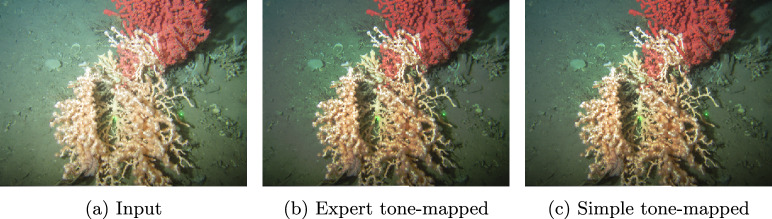
Example images results for the Gardline dataset

**Fig. 14:**
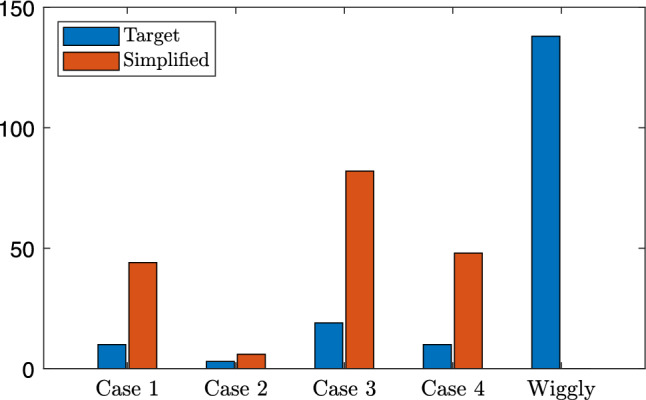
Classification of curve type from Gardline dataset before and after finding simple curve approximations with our method

We use the Gardline dataset as validation for our findings. Using the median expert $\Delta E$ colour difference, half of the simple images are less than 0.222 mean $\Delta E$, and the worst image is only 2.45. Figure 12 shows the tone curves for an example image from the dataset. Our method is applied to the blue curve describing the mapping from Fig.  13a–b . It yields the simple tone curve which is shown as the red dashed curve and produces the image in Fig. 13c . The expert tone-mapped image (13b) and our simple approximation (13c) are visually similar, and their colour difference is $\Delta E = 1.94$. We also report the curve cases in the original dataset and their simple counterparts in Fig. 14.

## Discussion

### Analysis of simple tone curves

**Fig. 15:**
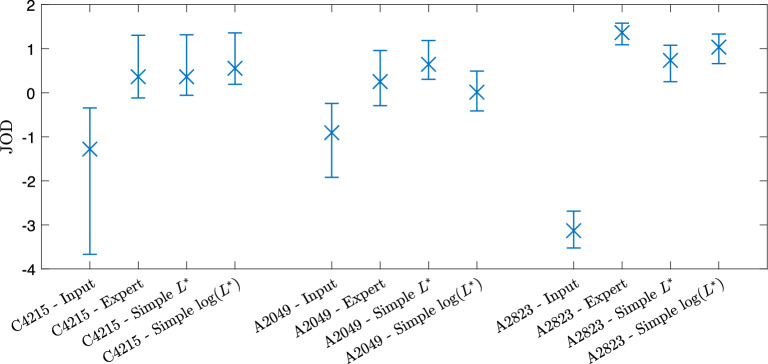
Psychometrically scaled JOD values and 95% confidence intervals for example images C4125, A2049, and A2823

Our aggregate results show that experts typically make simple tone adjustments. For the vast majority of cases where they do not, there is a simple tone curve that produces an equivalent enhancement. Here, we analyse each example individually, discussing the successes and limitations of the method. To aid in our discussion, Fig.  15 shows JOD values and confidence intervals for each rendition of three individual example images.

Figure 6 shows the enhancement of Expert C on image 4215 and is the 44th worst image as ranked by mean $\Delta E$ for $L^*$ methodology. Subplots (e) and (f) show that the expert’s curve clearly has multiple inflexion points and our simple approximations match the expert curve well. The mean $\Delta E$ differences between the simple and ground truth images are 1.62 and 2.01 for the $L^*$ and $\log (L^*)$ solution, respectively. Since the mean $\Delta E$ value is less than 3, we expect the images to be not readily noticeably different; we observe the images in (b), (c), and (d) are visually similar. Figure 15 shows similar JOD values for the three output renditions. The expert and simple $L^*$ renditions are identical (0.36) and $\log (L^*)$ is 0.55. The observers rated the renditions similarly and the relatively large confidence intervals mean observers voted inconsistently, which is further evidence that the renditions are visually very similar. This is an example where our simple tone curve solution approximates the expert tone curve well and the extra complexities of the expert’s curve contribute an insignificant difference to the image from a preference viewpoint.

In Fig. 7, the differences between $\hat{\mathcal {P}}$ and $\mathcal {P}^{G}$ are 3.12 and 2.14 for $L^*$ and $\log (L^*)$ methodologies, respectively. This example is the third worst result for of all 25,000 expert tone curves with the $L^*$ solution. The images are similar, but the differences are noticeable; however, the $\log (L^*)$ solved for curve appears slightly more similar to the ground truth. In the subjective evaluation experiment, the JOD values for each rendition are: (a) −0.91, (b) 0.25, (c) 0.65, (d) 0.01. The simple rendition solved for with the $L^*$ methodology is preferred by the observers against the other renditions. Naïvely, we might expect the expert ground truth rendition to be preferred when the simple rendition deviates from the ground truth. The implicit assumption is that the greater expressive power of a complex tone curve is required for a photographer to make a pleasing enhancement and if the simple curve does not have that expressive power, then it must be inferior. However, there is some evidence to suggest that simple curves may be preferred, for example, see Fig. 7.

Interestingly, in [17], underwater image analysts enhanced images that were then approximated by a Weibull tone map. The Weibull distribution is a unimodal function with two parameters, and the tone map is the cumulative sum of the distribution which will have a single inflexion point and is simple. The researchers conducted a pairwise comparison experiment and found that the analysts preferred the Weibull tone-mapped images over their often non-simple tone adjustments.

### Tone curves in visual processing

Many tone mapping operators employ compressive tone curves such as logarithmic or power law with no inflexion point, or sigmoidal S-shaped curves with an inflexion point. The human visual system operates in up to 12 orders of magnitude of radiance that must be compressed into a few orders using visual adaption and stimulus–response functions [38]. It does this so that our vision is sensitive to mid tone regions whilst highlights saturate and shadows are compressed. Perceptual tone mapping operators are based on these stimulus–response functions of the human visual system. For example, Stevens’ law [39] is a concave function with no inflexion point and the Naka Rushton model [40] is a S-shaped response with a single inflexion point; both widely used as a foundation for designing the shape of a tone curve. Our definition of a simple tone curve controls the shape of the tone curve by restricting to zero or one inflexion points. It offers more freedom than specifying a particular functional form yet maintains important shape characteristics. These models might correlate with the preference seen towards images enhanced with the simple tone curve over the expert’s complex enhancement.

### Failure Case Analysis

Let us return to the example illustrated in Fig.  8. The colour difference between simple and ground truth is 3.13 for the $L^*$ solution which is noticeable and 1.92 for the $\log (L^*)$ solution which is more similar to the ground truth but still with noticeable differences. Correlating with the visual results, the simple curve approximation in the log domain seems to better fit the tone curve data. The expert tone curve has two main inflexion points with some smaller deviations also. In subplot (e), the simple approximation of the expert curve fits the highlights well but does not fit as well in the shadows and mid tones. The foreground of the image in (c) is noticeably darker than (b) which is explained by this fitting. By contrast, the solution for the $\log (L^*)$ methodology fits well in the shadows and less well in the highlights, resulting in darker clouds in image (d) than (b).

In terms of subjective evaluation, the JOD values from Fig.  15 are (a) −3.13, (b) 1.36, (c) 0.73, (d) 1.03. In this example, the mean $\Delta E$ shows that the simple tone curve did not have enough expressive power to capture the photographer’s enhancement. Whilst this sometimes leads to a preferred rendition, observers deemed the complex adjustment necessary by preferring the expert’s ground truth rendition. The $\log (L^*)$ solution was more preferred than the $L^*$ solution by fitting to a different region of the tones, but the complex expert enhancement was most preferred.

Our optimisation method operates on the expert tone curve and finds the closest curve possible subject to the simple criteria. The approach has no knowledge of the image content or relative proportions of brightnesses in the image. This allows the method to be fast, but can be a limitation where closer fitting in another region of the tones produces a closer visual match.

Figure 9 shows the enhancement of image 3123 by expert C. The expert curve is almost simple, but there are many small deviations which induce inflexion points. The $L^*$ methodology simple solution is very close to the expert curve and produces images that are a very close visual match where the differences to ground truth are almost certainly imperceptible (mean $\Delta E = 0.0406$). However, the $\log (L^*)$ solution fails on this example, producing the fourth worst mean $\Delta E = 5.60$. Solving in the $\log (L^*)$ encoding means the logarithmic function is applied to both axes which stretches the proportion of the domain that is attributed to dark values. The curve shape in the dark region is exaggerated, which in this case amplifies the small deviations that were minor in $L^*$ encoding. In turn, greater emphasis is given to the dark values and the $\log (L^*)$ solution fits to inflexion points that were small in the L* and in this case does not fit well to the mid tones and highlights which have a large impact on the visual appearance of the image.

**Fig. 16:**
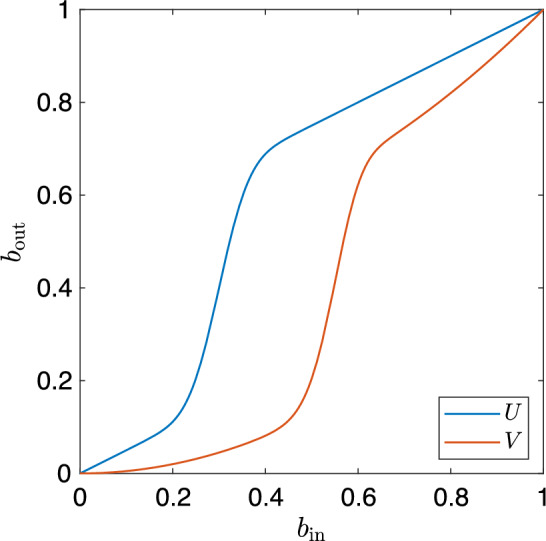
Visualisation of a simple function composed with a gamma function where an inflexion point is introduced

**Fig. 17:**
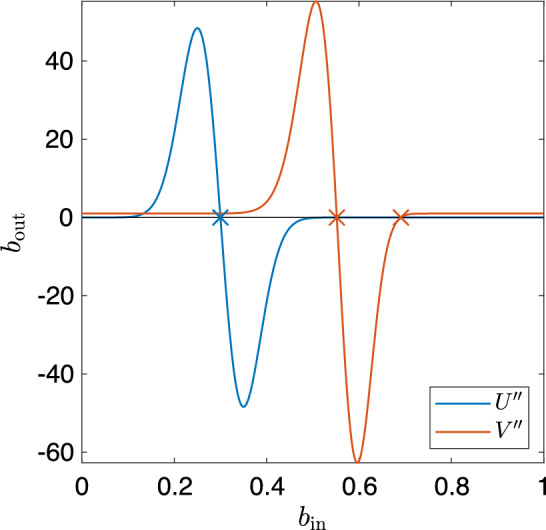
Second derivatives of function in Fig.  16 with zero crossings marked

**Fig. 18:**
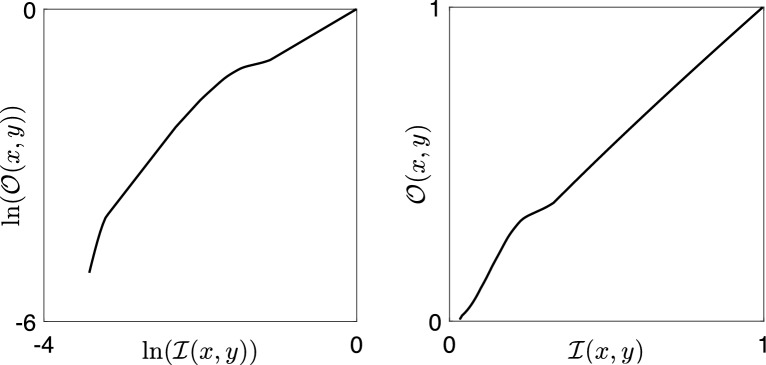
The tone curve in the left plot is simple in the log domain, but the same curve is not simple in the right plot in the primal domain

We have shown that experts generally make simple enhancements. Where they do not, except for a handful of examples, they are well approximated by a simple tone curve. In some cases, simple tone curves are preferred over an expert’s complex enhancement, which is supported by other studies and theoretical foundation. This begs the question as to whether photo-processing tools should allow (or at least make it easy) for users to make non-simple tone adjustments. It could be that they are allowing their users to make adjustments that will not be preferred or, perhaps, as preferred as a simple adjustment.

### Simplicity under transformation

Here we consider the question of whether a simple curve remains simple under composition with an encoding function. We find, by stepping through a specific example, that this is not the case. An implication of this result is that simple curves applied in one domain do not necessarily remain simple in a transformed domain.

One transformation frequently used in camera processing pipeline is the power law to apply a gamma curve. For example, applying (approximately) 1/2 gamma to condition a linear image for display (since displays have a squared transfer function). In itself, the gamma function can be considered a tone curve, but we can also use it to transform the space of a tone curve using the composition of functions. Let us investigate how a tone curve *works* when a gamma is applied to it. Let us consider the encoding function,

31 $$\begin{aligned} g: b \mapsto b^ \gamma \end{aligned}$$

where $\gamma =2$. Next we define an S-shaped (because these are the most commonly used in image enhancement) tone curve $b_\textrm{out} = U(b_\textrm{in})$ which is the sum of a linear scaling function and the integral of the normal distribution.

32 $$\begin{aligned} U(b_\textrm{in}) = \alpha \left( \beta b_\textrm{in} + \int _{-\infty }^{b_\textrm{in}} \phi (x; \mu , \sigma ) \, \textrm{d}x \right) , \end{aligned}$$

where

33 $$\begin{aligned} \phi (x; \mu , \sigma ) = \frac{1}{\sigma \sqrt{2 \pi }} \textrm{e} ^{- \frac{(x-\mu )^2}{2\sigma ^2}}. \end{aligned}$$

This tone curve function is plotted in blue in Fig.  16 where $\alpha =0.5, \beta =1, \mu =0.3, \sigma =0.05$ and we see the curve is simple.

Now, let us apply gamma to the input image by applying our encoding function *g* to the input brightnesses. This yields a new tone curve function,

34 $$\begin{aligned} V(b_\textrm{in}) = U(g(b_{in})) = \alpha \left( \beta b_{in}^\gamma + \int _{-\infty }^{b_{in}^\gamma } \phi (x; \mu , \sigma ) \, \textrm{d}x \right) . \end{aligned}$$

This is also shown in 16, plotted in red. Looking closely, we see *U* has a single inflexion point, but *V* which is the composition with gamma has two inflexion points. Recalling that an inflexion point occurs at the zero crossing of the second derivative, we solve for the second derivatives of *U* and *V*. These are plotted in 17, with $''$ denoting the second derivative. Visual analysis shows $U''$ has a single zero crossing and analytical analysis shows $U''(0.3) = 0$. We find, using a numerical solver, that $V''= 0$ at brightnesses $0.552 \; \textrm{and} \; 0.691$ (to 3 decimal places). By example, we have shown that a simple tone curve composed with another function may not always remain simple.

In Fig. 18, we show a curve that is simple in the log domain and is not simple in the primal domain.

## Conclusion

Tone mapping is a very powerful technique for image enhancement and is a key part of the toolset in photo editing software as well as being implemented in every photographic camera pipeline. In this paper, we considered whether tone mappings made by users are simple or complex. Simple tone curves were defined to be monotonically increasing curves that either have no or one inflexion point. Conversely, complex curves are those that cannot be simply defined. A computational method is presented to find the best simple tone curve that approximates a complex tone mapping. Experiments conducted on two datasets comprising, respectively, 25,000 and 180 manually tone-adjusted images found that the tone adjustments made were either simple or that they could, using an objective similarity measure, be well approximated by a simple curve adjustment. Human observer preference experiments showed simple curves produce equally preferred images to photographers, often non-simple, adjustments. In fact, for tone curves calculated in $\log (L^*)$ space, we found that simple tone curve adjustments were slightly preferred.

## Data Availability

The MIT-Adobe FiveK dataset is available at https://doi.org/10.1109/CVPR.2011.5995413. The anonymised data collected from the psychophysical pairwise preference experiment are available from the corresponding author on reasonable request. The Gardline dataset was used under licence and is not publicly available.
